# Integrated bioinformatics identifies ferroptosis biomarkers and therapeutic targets in idiopathic pulmonary arterial hypertension

**DOI:** 10.1038/s41598-025-11066-y

**Published:** 2025-07-12

**Authors:** Yuhao Zhang, Tao Qian, Wei Jiang, Haoyong Yuan, Ting Lu, Ni Yin, Zhongshi Wu, Can Huang

**Affiliations:** 1 Department of Cardiovascular Surgery, The Second Xiangya Hospital, Central South University, Changsha, 410011 Hunan China; 2 Engineering Laboratory of Hunan Province for Cardiovascular Biomaterials, Changsha, 410008 Hunan China

**Keywords:** Potential biomarkers, Therapeutic targets, Ferroptosis, Idiopathic pulmonary hypertension, Computational biology and bioinformatics, Drug discovery, Genetics, Immunology

## Abstract

Idiopathic pulmonary arterial hypertension (IPAH), a rare and devastating pulmonary vascular disorder, is characterized by cellular proliferation and vascular remodeling. Although previous studies have underscored that ferroptosis, an iron-dependent cell death process, plays an important regulatory role in pulmonary artery hypertension, its role remains understudied. Gene expression profiles were downloaded from the Gene Expression Omnibus(GEO). Differentially expressed genes (DEGs) were screened using R software and intersected with a ferroptosis database (FerrDb V1) to identify ferroptosis-related DEGs. GO and KEGG analyses were performed to explore biological functions and potential pathways. LASSO and SVM-RFE algorithms were used to identify optimal gene biomarkers for IPAH. GSVA and GSEA were conducted to explore biological functions and potential pathways associated with these biomarkers. The CIBESORT software was employed to predict immune genes and functions. Of 237 ferroptosis-related genes (FRGs), 27 differentially expressed FRGs (DE-FRGs) showed significant differences between IPAH and normal samples in GSE48149, with 15 downregulated and 12 upregulated genes. Six DE-FRGs, including *KEAP1*, *TNFAIP3*, *MEG3*, *NFS1*, *PRDX1*, and *BEX1*, were identified as predictive diagnostic genes for IPAH. Among these DE-FRGs, *PRDX1* and *TNFAIP3* were the most promising diagnostic genes for IPAH and may play a corresponding role in IPAH by participating in the cell cycle, lysosomes, immune response, vascular smooth muscle contraction, and various diseases. CIBERSORT analysis revealed a positive correlation between neutrophils and *TNFAIP3*, whereas macrophages M0 exhibited a negative correlation with *PRDX1*. Through comprehensive bioinformatics analysis, we identified six differentially expressed ferroptosis-related genes (DE-FRGs)—*KEAP1*, *TNFAIP3*, *MEG3*, *NFS1*, *PRDX1*, and *BEX1*—in idiopathic pulmonary arterial hypertension (IPAH). Among these, *PRDX1* and *TNFAIP3*, which play key roles in IPAH pathogenesis, emerged as the most promising diagnostic biomarkers.

## Introduction

Idiopathic pulmonary arterial hypertension (IPAH) is a rare and devastating pulmonary vascular disorder characterized by persistently elevated pulmonary artery pressure, often progressing to right ventricular hypertrophy and heart failure, and it has a relatively low estimated prevalence of 5.9 cases per 1 million individuals^1,2^. Despite treatment progress, the prognosis of newly diagnosed patients with IPAH remains suboptimal, with 5- and 10-year transplant-free survival rates of 71% and 35%, respectively^3^. IPAH exhibits intricate pathophysiological alterations driven by genetic, epigenetic, and environmental factors, which result in proliferative remodeling, endothelial dysfunction, fibrosis, inflammation, and cancer-like attributes^4–6^. Although mutations in genes such as *BMPR2*,* ATP13A3*,* AQP1*,* ABCC8*,* KCNK3*,* SMAD9*,* Sox17*,* CAV1*,* TBX4*, *and KDR* have been established as biomarkers of IPAH, the exact pathogenesis of this disease remains unclear^7^. Whereas the principles of predictive, preventive, and personalized medicine (PPPM) have been successfully applied in oncology to screen for cancer biomarkers and identify therapeutic targets through genome sequencing^8^, their application in the realm of IPAH is conspicuously lacking in terms of early prediction and preventative strategies. In fact, PPPM has the potential to expedite diagnosis, alleviate emotional uncertainty in patients, reduce the burden on healthcare resources, and enable treatment initiation at an earlier stage, when interventions are typically more effective^9^. Therefore, the expedient identification of robust molecular markers is essential for improving the treatment and diagnosis of IPAH.

Iron deficiency is a common occurrence in 43–63% of patients with IPAH and is associated with decreased exercise tolerance and elevated mortality rates^10,11^, indicating that iron metabolism is crucial in IPAH. Ferroptosis, a regulator of cell death characterized by iron-dependent lipid peroxidation and oxidative stress, is involved in various pathological conditions, including drug-induced liver damage, acute kidney injury, cancer, neurodegenerative disorders, and cardiovascular disease^12^. Previous research has suggested that ferroptosis is activated and contributes to pulmonary artery hypertension (PAH)^13^. Dai et al. showed that ferroptosis in pulmonary artery endothelial cells, mediated by peroxiredoxin 6 (PRDX6), plays a role in the development of monocrotaline-induced pulmonary hypertension (MCT)^14^. For pulmonary artery endothelial ferroptosis, in which the NLRP3 inflammasome is activated via the HMGB1/TLR4 pathway in MCT-affected rats, Lan et al. suggested that a ferroptosis inhibitor could be a novel therapeutic target for PAH^15^. Moreover, Sun et al. showed that increased levels of NDUFA4L2 are crucial in advancing hypoxia-induced pulmonary hypertension by promoting ROS production and pulmonary arterial smooth muscle cell (PASMC) proliferation, suggesting that targeting NDUFA4L2 could be a promising novel therapeutic approach for PAH^16^. Furthermore, Hu et al. demonstrated that SLC7A11 suppresses ferroptosis and enhances proliferation in PAH, effectively rebalancing the dynamics between cell death and proliferation in PASMCs^13^. Additionally, bioinformatic evidence suggests the coexistence of both activation and silencing of ferroptosis in PAH^17^. In fact, existing studies present some paradoxical phenomena, and the specific mechanism by which ferroptosis aggravates PAH remains unknown^18^, emphasizing the need for further research in this area.

We thus hypothesize a robust association between ferroptosis-related genes (FRGs) and IPAH, encompassing its intricate immune microenvironment. Our research elucidated the pivotal functional roles of FRGs in accurately predicting IPAH prognosis, providing novel insights into the mechanisms underlying immune regulation within the IPAH patient cohort. The exploration of FRGs as prospective biomarkers for IPAH has the potential to empower high-risk subpopulations with the means to modulate their prognosis via personalized medical interventions firmly situated within the paradigm of PPPM.

## Experimental procedure

### Data source

We downloaded gene expression profiles and FRGs from the Gene Expression Omnibus (GEO) (https://www.ncbi.nlm.nih.gov/geo/*)* and the FerrDb online database (FerrDbV1, 2020) (http://www.zhounan.org/ferrdb/*)*, respectively^19^. GSE48149, consisting of eight IPAH and nine healthy tissue samples, was designated as the training set. Validation sets, including GSE117261 (31 IPAH and 25 healthy tissue samples), GSE84395 (11 IPAH and 18 healthy pulmonary endothelial cells [PEC] samples), GSE33463 (30 IPAH and 41 healthy peripheral blood mononuclear cell [PBMCs] samples), and GSE169471 (3 IPAH and 8 healthy tissue samples), were used to confirm marker gene levels. The Drug Gene Interaction Database (DGIdb, https://www.dgidb.org), and Cytoscape software were used for predicting potential drugs or molecular compounds interacting with marker genes and visualizing the drug–gene interaction network, respectively^20,21^.

### Identification of differentially expressed genes (DEGs)

The expression profiles of 237 FRGs in normal and IPAH samples were extracted from the GSE48149 database. Subsequently, the “limma package” in R software (R version 4.2.2) was employed to identify significant DE-FRGs between the two groups using parameters |Log2fold change| >0.5 and adj.*P* < 0.05 (Table 1)^22^. The “pheatmap and ggplot2 packages” were used for constructing the heatmap of DEGs, and the “Corrplot package” was used for analyzing the correlation between DE-FRGs, with *p* < 0.05 considered significant^23^.

**Table 1:** Twenty-seven DE-FGRS of 237 FRGs with 15 down-regulated and 12 up-regulated genes in GSE48149.

DE-FGRS	Con Mean	Treat Mean	*P*-value	Type
TF	7.694373	8.4974365	0.00370218	Up
PGD	13.04663944	12.1850975	0.000575895	Down
TP53	8.520795111	8.042711125	0.000329083	Down
KEAP1	10.24850689	9.823452	0.000164541	Down
ULK2	7.649207444	7.845423875	0.007897984	Up
SOCS1	9.056795444	9.6826355	0.00246812	Up
MAPK14	7.900324556	7.597012875	0.001563143	Down
IFNG	7.208926333	9.201221875	0.000575895	Up
TNFAIP3	10.645038	12.82404025	0.000329083	Up
IDH1	12.21267922	11.32308813	0.00370218	Down
FADS1	11.09215022	9.91926825	0.000164541	Down
SLC25A28	11.96804178	12.51708963	0.000987248	Up
MFN2	10.87829122	10.52674388	0.007897984	Down
SMPD1	8.782253111	8.43587875	0.00246812	Down
USP11	9.837170778	10.26258975	0.000575895	Up
FADS2	8.553869778	7.924775875	0.00370218	Down
ADAM23	7.774517222	7.343884	0.005512135	Down
MEG3	9.803914889	10.81344388	0.000082270	Up
NQO1	10.31008733	9.484728375	0.000987248	Down
SLC3A2	10.94148889	10.162668	0.000987248	Down
SRC	9.538373889	9.81226825	0.007897984	Up
NFS1	8.638115444	9.236236375	0.007897984	Up
ENPP2	10.79377567	12.20948913	0.000164541	Up
CBS	10.45458022	9.443824375	0.000987248	Down
SREBF1	9.900411	9.25557975	0.00246812	Down
AHCY	12.02612433	11.47570063	0.000575895	Down
VDR	7.946485444	7.618824375	0.007897984	Down
PRDX1	13.97259856	13.466841	0.002421774	Down
COPZ1	11.71979467	11.35591175	0.00246812	Down
PARP10	9.291106333	9.657784	0.00370218	Up
PARP14	10.75779833	11.2985625	0.007897984	Up
BEX1	8.504673667	9.509029125	0.000987248	Up
MGST1	11.86885689	10.62353375	0.000164541	Down
DHODH	7.824816333	7.38555675	0.000575895	Down
PDK4	11.468836	13.4470235	0.000987248	Up
MAPKAP1	9.484097	9.0442895	0.002421774	Down
TERT	7.168574111	7.04489325	0.00246812	Down

### Functional enrichment analysis

The “clusterProfiler package” was employed to perform the Gene Ontology (GO) and Kyoto Encyclopedia of Genes and Genomes (KEGG) enrichment analyses of DE-FRGs. GO analysis explored biological functions in three categories: biological process (BP), cellular component (CC), and molecular function (MF). Simultaneously, KEGG analysis was used to explore potential pathways^24–26^.

### Identification of optimal gene biomarkers for IPAH

The “glmnet package” and “e1071 package” were used to identify IPAH-related gene biomarkers using the least absolute shrinkage and selection operator (LASSO) and support vector machine-recursive feature elimination (SVM‐RFE) algorithms, respectively. The “Jvenn package” was employed to construct the Venn diagram. Overlapping biomarkers are considered the most favorable for IPAH^27^. Receiver Operating Characteristic (ROC) curves, Area Under the Curve (AUC), sensitivity, specificity, and accuracy were calculated to evaluate the diagnostic ability of the selected gene markers. The “glm package” was used to construct a logistic regression model based on the six marker genes to predict GSE48149 dataset sample types and test the diagnostic value of the model using ROC curves^28^.

### Single-gene gene set enrichment analysis (GSEA)

The “GSEA package” was employed to explore pathways or biological functions enriched by those six marker genes. After calculating the associations between the marker genes and all other genes in the GSE48149 dataset, all genes were sorted according to their high-to-low correlations and listed in the gene set for analysis. Meanwhile, the KEGG or Go set was invoked as a predefined set to detect its enrichment in the gene set.

### Single-gene gene set variation analysis (GSVA)

The GSVA (version 1.38.0) package in R was used for this analysis^29^. The KEGG pathway set was adopted as the background gene set for the GSVA of diverse marker genes. Simultaneously, the “limma package” was used to analyze the difference in GSVA score of the marker gene’s high- and low-expression group samples based on the significance levels of |t| >2 and *p* < 0.05. The pathway was activated in the high-expression group when t > 0; otherwise, it was activated in the low-risk group.

### Immune infiltration analysis

CIBERSORT characterizes the cellular composition of complex tissues based on gene expression data^30^. The GSE48149 dataset was analyzed using CIBERSORT software to estimate the proportions of 22 types of infiltrating immune cells. Additionally, immune genes and functions in each tissue were predicted using CIBESORT software.

### mRNA–miRNA–lncRNA CeRNA network construction

The construction of a ceRNA network involves numerous lncRNAs, miRNAs, and mRNAs, along with their interactions. We predicted the binding of mRNA–miRNA nucleic acids using TargetScan, miRDB, and miRanda databases^31–33^. Furthermore, miRNA–lncRNA binding interactions were identified using SpongeScan^34^ to obtain the mRNA–miRNA–lncRNA ceRNA network.

### Validation of marker genes in a single dataset

#### Single-cell RNA sequencing (scRNA-seq) data analysis

scRNA-seq data, including three IPAH samples and eight control samples, were downloaded from the GEO database (GSE169471). The UMI count matrix was imported into R to generate Seurat objects using the “Seurat package.” Genes detected in fewer than three cells and cells with fewer than 200 genes were excluded after quality control. The SCTransform function was applied for data normalization, variable feature detection, and data scaling. Subsequently, the FindIntegrationAnchors and IntegrateData functions were used to identify anchors for dataset integration, eliminating batch effects between samples. Principal component analysis was conducted for dimensionality reduction using the RunPCA function, and the top 30 principal components were selected to construct a common nearest-neighbor graph in low-dimensional subspaces. Cell clustering was executed with the FindClusters function, setting the resolution to 0.4, and nonlinear dimensionality reduction was performed using the RunUMAP function.

#### Cell type identification and gene expression analysis

Cell types were annotated based on the expression of canonical gene markers in the lung tissue from previously published articles and assigned to the corresponding clusters. Expression analysis of target genes was presented as dot, violin, box, and feature plots using functions from the Seurat and ggplot2 packages.

### Statistical analysis

All statistical analyses were performed using R software (V4.2.2). The Wilcoxon rank-sum test was used to compare the two groups, and Pearson’s correlation analysis was used to evaluate the relationship between the DE-FRGs. Statistical significance was set at *p* < 0.05.

## Results

### Identification of DE-FRGs from GSE48149

**Fig. 1:**
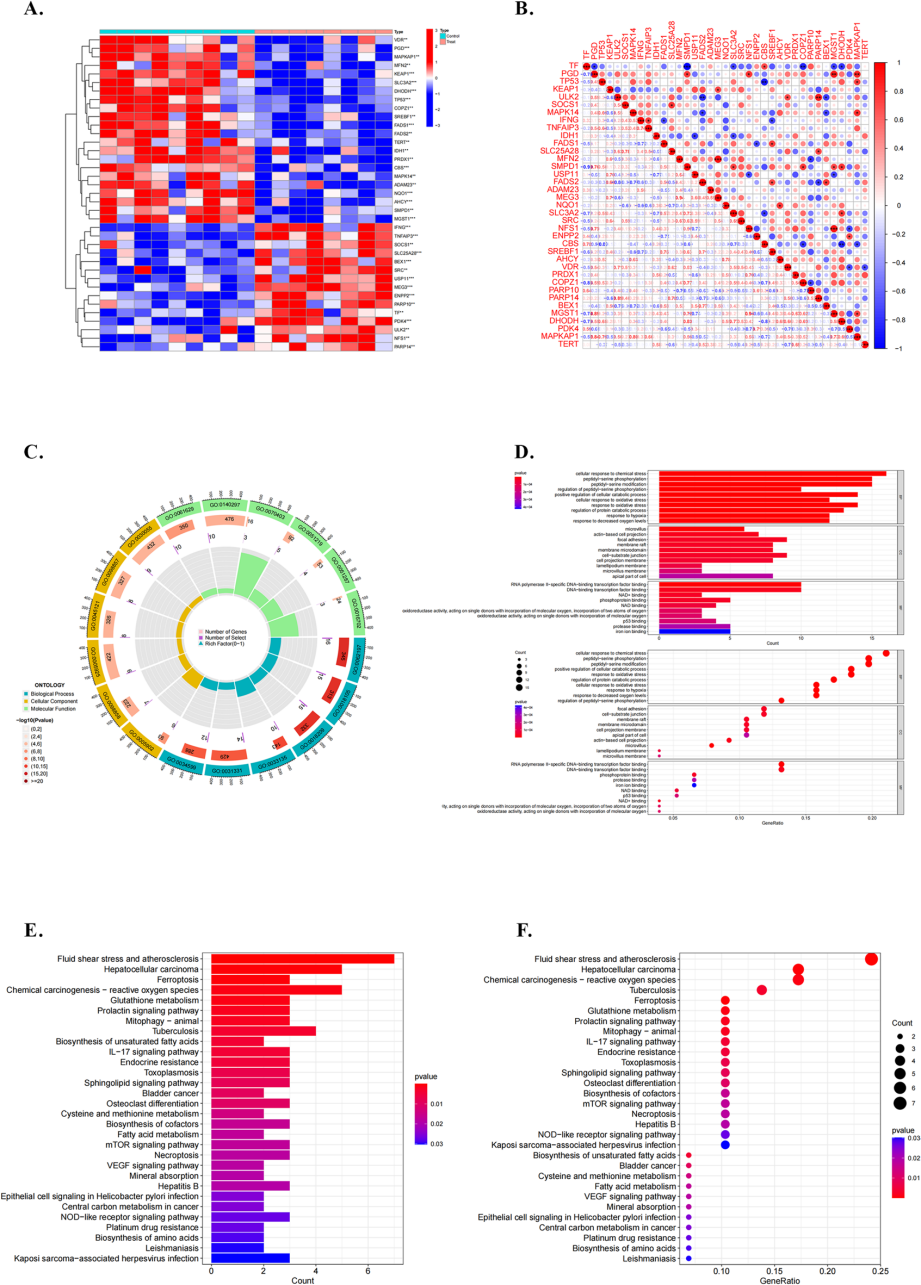
Identification of DE-FRGs from the GSE48149. **(A)** Visualization of 15 downregulated and 12 upregulated genes between IPAH and normal samples using a heatmap. **(B)** Associations between DE-FRGs, with no correlation between *ADAM23* and *SRC* and any other DE-FRGs. **C** and **D**. GO enrichment and Reactome pathway analyses were employed to elucidate DE-FRG-associated biological functions and pathways. **E** and **F**. Reactome pathway analyses revealed remarkable enrichment in fluid shear stress, atherosclerosis, hepatocellular carcinoma, and chemical carcinogenesis reactive oxygen species.

Of 237 FRGs, 27 DE-FRGs exhibited a significant difference between IPAH and normal samples in GSE48149, with 15 downregulated and 12 upregulated genes (Table 1), visualized using a heatmap (Fig. 1A). The correlation plots demonstrated an association among DE-FRGs, with no correlation between *ADAM23* and *SRC* and any other DE-FRGs (Fig. 1B).

### Functional annotation for DE-FRGs

GO enrichment and Reactome pathway analyses were conducted to elucidate the biological functions and pathways associated with DE-FRGs. GO enrichment analyses revealed significant associations of DE-FRGs with CC such as “focal adhesion,” “cell–substrate junction,” and “membrane raft.” BP annotation indicated a notable relation of DE-FRGs with “cellular response to chemical stress,” “cellular response to peptidyl-serine phosphorylation,” and “peptidyl-serine modification.” Additionally, MF annotation suggested a close association between DE‐FRGs and “RNA polymerase II-specific DNA-binding transcription factor binding,” “DNA-binding transcription factor binding,” and “phosphoprotein binding”(Fig. 1C and D). Reactome pathway analyses revealed marked enrichment in pathways such as fluid shear stress, atherosclerosis, hepatocellular carcinoma, and chemical carcinogenesis reactive oxygen species (Fig. 1E and F). Based on this evidence, DE‐FRGs may play a crucial role in IPAH by participating in the regulation of autophagy, cytokines, kinases, and immune cells.

### Identification of six DE-FRGs as diagnostic genes for IPAH

**Fig. 2:**
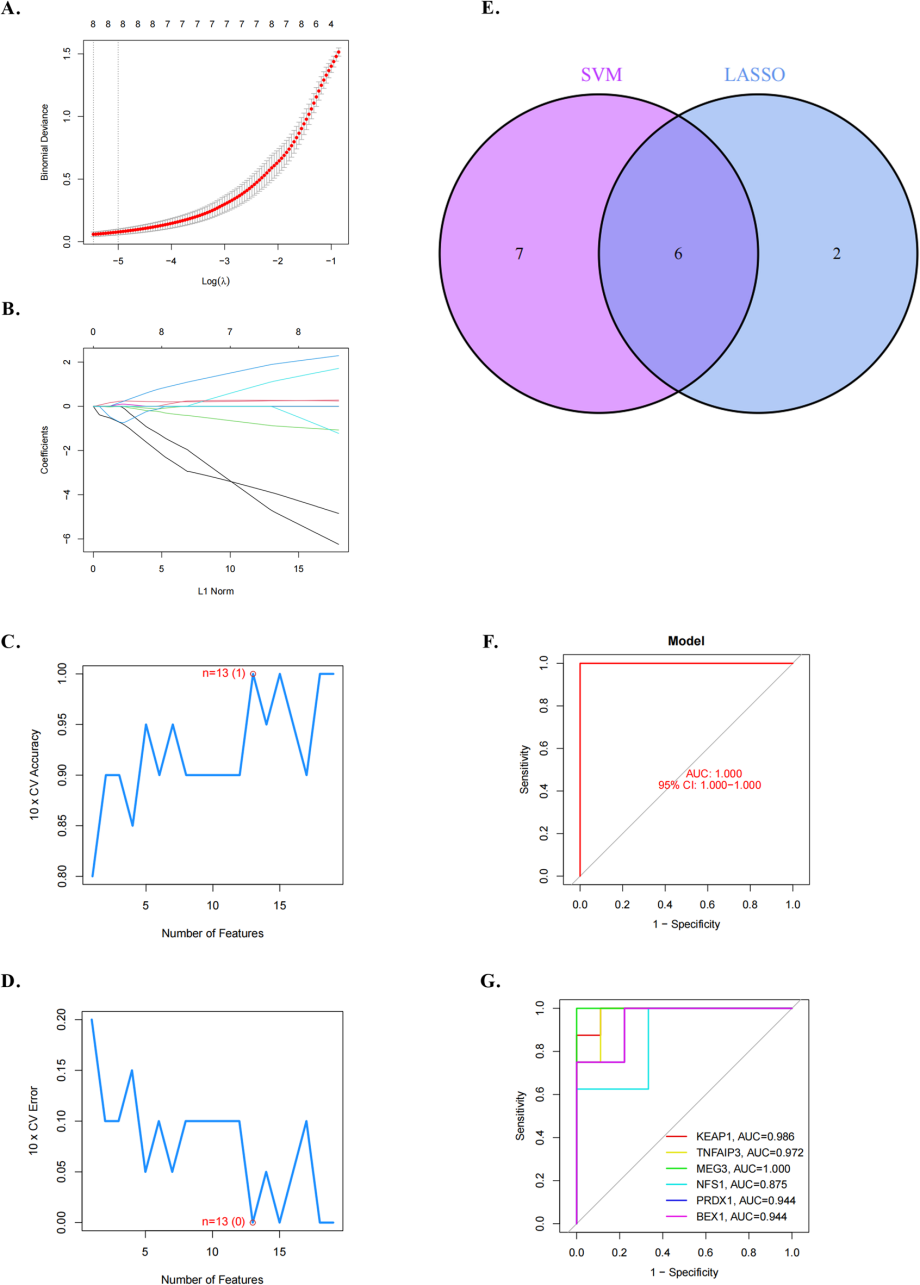
LASSO and SVM-RFE were used to screen for significant DE-FRGs to distinguish patients with IPAH from normal individuals in the GSE48149 dataset. **A** and **B**. Eight IPAH-related features were selected using LASSO logistic regression with penalty parameter tuning by 10-fold cross-validation. **C** and **D**. Twenty-seven DE-FRGs were filtered to identify the best feature gene combination using the SVM‐RFE algorithm. Finally, 13 genes (minimal RMSE = 0 and maximal accuracy = 1) were identified as the best feature genes. **E**. Marker genes were obtained from the LASSO and SVM-RFE models. **F**. A logistic regression model was used to identify the AUC of the disease samples. **G**. ROC curves for the six marker genes.

To assess the potential utility of predicted DE-FRGs as diagnostic genes for IPAH, we explored variations between patients with IPAH and healthy individuals. Initially, two machine learning algorithms (LASSO and SVM-RFE) were employed to identify significant DE-FRGs capable of distinguishing patients with IPAH from normal individuals in the GSE48149 dataset. Using LASSO logistic regression with penalty parameter tuning through 10-fold cross-validation, eight IPAH-related features were selected. The SVM-RFE algorithm further filtered 27 DE‐FRGs to identify the optimal gene combination, resulting in the identification of 13 genes (minimal RMSE = 0 and maximal accuracy = 1) as the best feature genes. Intersection of marker genes from LASSO and SVM-RFE models led to the identification of six DE‐FRGs—*KEAP1*, *TNFAIP3*, *MEG3*, *NFS1*, *PRDX1*, and *BEX1—*as potential diagnostic genes for IPAH. These genes were then used in subsequent analyses. A logistic regression model, implemented using the R “glnn package” based on the six marker genes, achieved an AUC value of 1, indicating perfect discriminatory ability. Subsequently, an ROC curve was generated to illustrate the efficacy of the six marker genes in distinguishing patients with IPAH from normal individuals, with AUC values exceeding 0.8 for all genes. Consequently, our logistic regression model demonstrated superior accuracy and specificity compared to individual marker genes. Further analysis revealed distinct expression patterns in IPAH tissues relative to normal tissues, with decreased expression levels of *KEAP1* and *PRDX1* and increased expression of *BEX1*, *MEG3*, *NFS1*, and *TNFAIP3* in IPAH tissues (Fig. 2).

### Marker gene associations with IPAH-related pathways

**Fig. 3:**
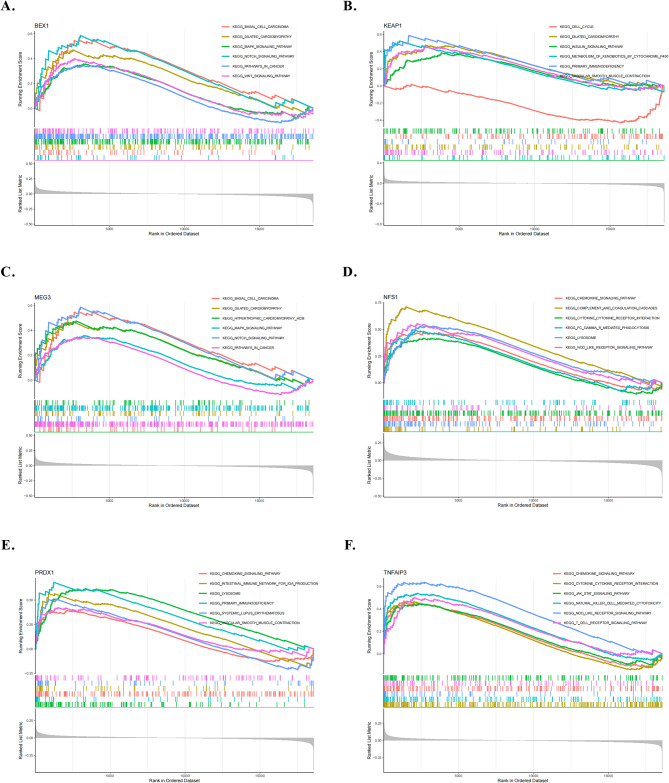
Single-gene GSEA–KEGG pathway analysis was employed to explore the potential function of marker genes in distinguishing IPAH from normal controls: **A**. *BEX1*, **B**. *KEAP1*, **C**. *MEG3*, **D**. *NFS1*, **E**. *PRDX1*, **F**. *TNFAIP3*.

We utilized single-gene GSEA-KEGG pathway analysis to explore the potential roles of marker genes in distinguishing patients with IPAH from normal controls, highlighting six pathways linked to these markers. Significantly, the marker genes demonstrated associations with the cell cycle, lysosomes, immune response (including natural killer cell-mediated cytotoxicity, T cell receptor signaling pathway, cytokine-receptor interaction, complement, intestinal immune network for IgA production, and Fc gamma R-mediated phagocytosis), vascular smooth muscle contraction, and various diseases (primary immunodeficiency, carcinoma, dilated cardiomyopathy, hypertrophic cardiomyopathy, and systemiclupuserythematosus). Furthermore, we observed tight associations of marker genes with xenobiotic metabolism by cytochrome p450, chemokine signaling pathway, MAPK signaling pathway, Nod-like receptor signaling pathway, Notch signaling pathway, WNT signaling pathway, JAK-STAT signaling pathway, and insulin signaling pathway (Fig. 3).

**Fig. 4:**
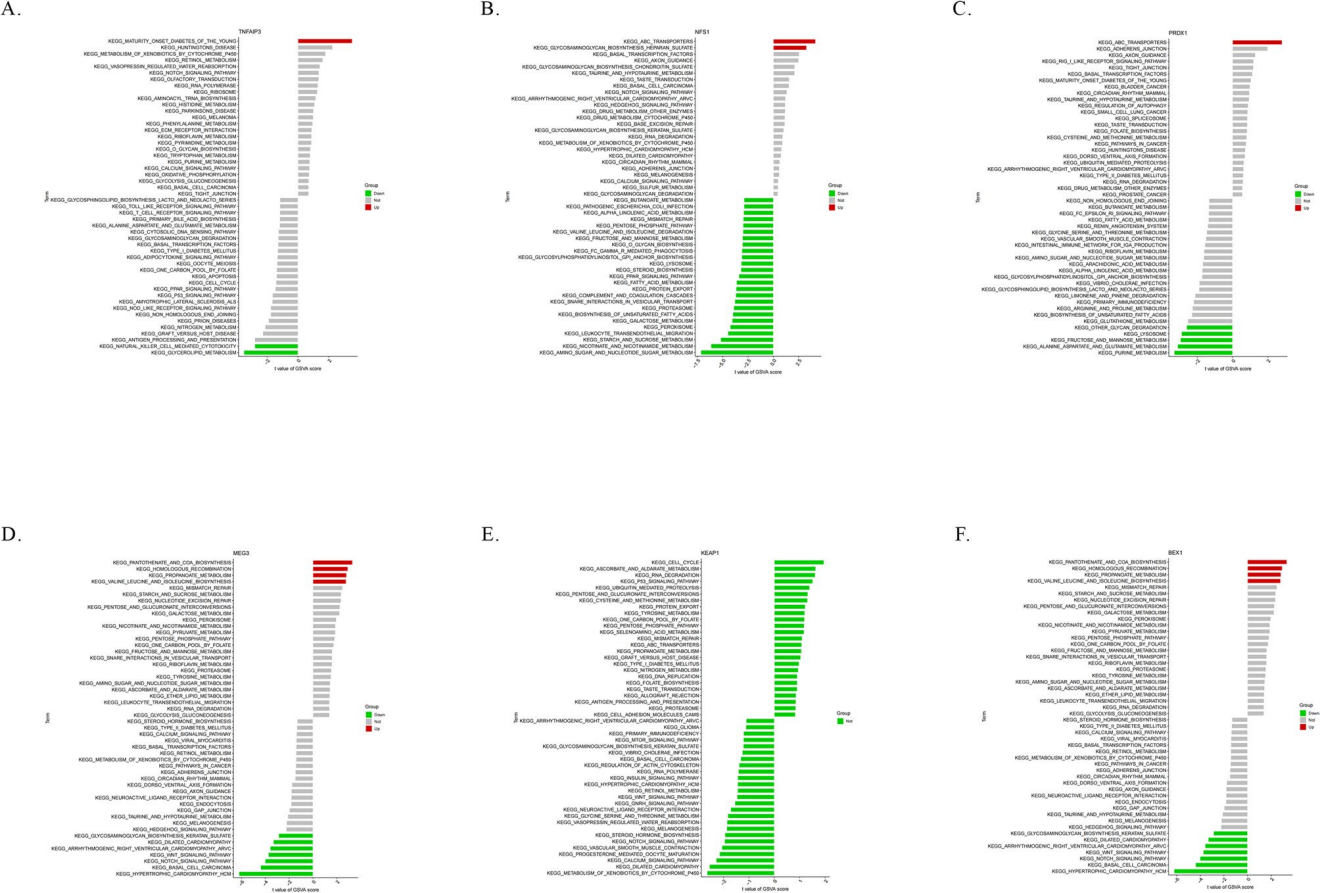
The differential activation levels of the pathway were analyzed between IPAH and normal controls according to GSVA and marker gene expression levels: **A**. *TNFAIP3*, **B**. *NFS1*, **C**. *PRDX1*, **D**. *MEG3*, **E**. *KEAP1*, **F**. *BEX1*.

Differential pathway activation levels were analyzed between patients with IPAH and normal individuals based on GSVA and marker gene expression levels (Fig. 4). Notably, *PRDX1* downregulation was implicated in IPAH pathogenesis by activating pathways such as “purine metabolism”, “alanine aspartate and glutamate metabolism”, “fructose and mannose metabolism”, “lysosome”, “other glycan degradation” and “other glycan degradation”, whereas its overexpression activated “ABC_transporters”. *TNFAIP3* upregulation was associated with the activation of “maturity onset diabetes of the young”, whereas its downregulation correlated with “glycerolipid metabolism” and “nature killer cell mediated cytotoxicity”. In addition, *MEG3* overexpression activated pathways such as “pantothenate and coa biosynythesis”, “homologous recombination”, “propanoate metabolism” and “valine leucine and isoleucine biosynthesis”, whereas its downregulation activated pathways such as “glycosaminoglycan biosynthesis keratan sulfate”, “dilated cardiomypathy”,“arrhythmogenic right ventricular cadiomyopathy”, “WNT signaling pathway”, “NOTCH signaling pathway”, “basal cell carcinoma” and “hypertrophic cardiomyopathy”. Moreover, *BEX1* upregulation was associated with “pantothenate and coa biosynthesis”, “homologous rcombination” and “propanoate metabolism,” whereas its downregulation may activate “arrhythmogenic right ventricular cadiomyopathy”, “dilated cardiomypathy”, “glycosaminoglycan biosythesis keratan sulfate”, “hypertrophic cardiomyopathy”, “WNT signaling pathway” “NOTCH signaling pathway” and “basal cell carcinoma”. *NFS1* downregulation significantly activated “starch and sucrose metabolism”, “nicotinate and nicotinamide metabolism” and “amino sugar and nucleotide sugar metabolism,” whereas its overexpression was associated with “ABC_transporters” and “glycosaminoglycan biosynthesis_heparan sulfate”. Interestingly, no significant difference in pathway activation levels of *KEAP1* was observed between the two groups.

### Immune microenvironment analysis

**Fig. 5:**
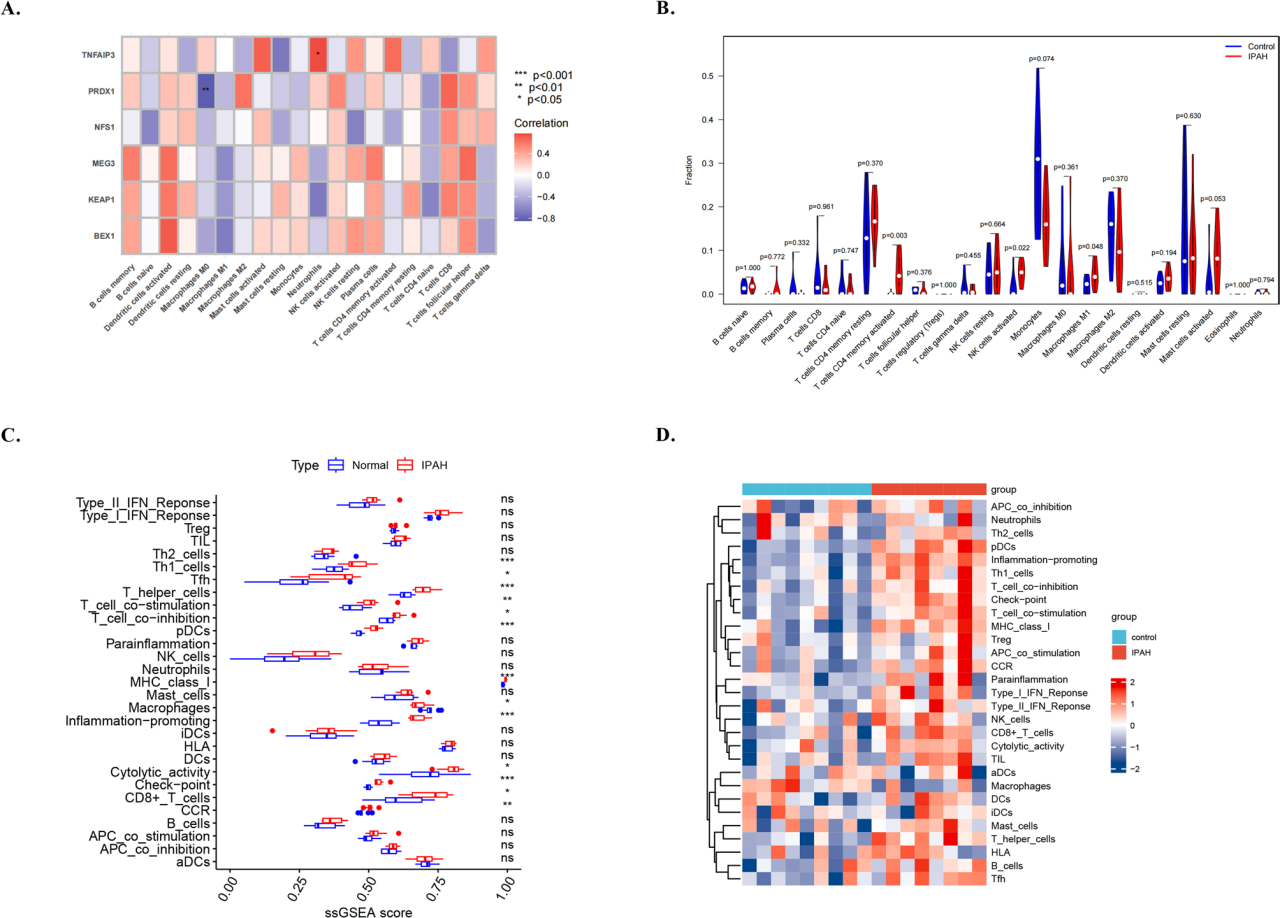
Immune microenvironment analysis: **A**. Pearson’s correlation analysis showing positive correlation between neutrophils and *TNFAIP3* and negative correlation between macrophages M0 and *PRDX1*. **B**. The CIBERSORT algorithm was employed to investigate the differences in the immune microenvironment between IPAH and normal controls. **C**. ssGSEA was used to calculate the difference in immune cell infiltration between the IPAH and normal control groups. **D**. Heatmap showing the differences in immune cell infiltration.

Based on the results, the marker genes were closely associated with immunity. Previous research has confirmed the close relationship between the immune response and IPAH^35^. The CIBERSORT algorithm was employed to investigate differences in the immune microenvironment between patients with IPAH and normal individuals. We observed overexpression of activated NK cells, macrophages M1, and CD4 memory T cells in patients with IPAH compared with normal individuals. Pearson’s correlation analysis suggested a positive correlation between neutrophils and *TNFAIP3*, whereas macrophages M0 showed a negative correlation with *PRDX1*. Subsequently, we compared different immune-related genes (IRGs) and functions between IPAH and normal samples. Immunostimulatory genes, including *TNFSF9*, *LDHA*, *JAK1*, and *FGL1*, were highly expressed in normal samples, whereas *LAG3*, *IFNG*, and *CTLA4* were upregulated in patients with IPAH. We compared the different immune functions in IPAH and normal controls and found that most immune functions, including Th1_cells, Tfh, T_helper_cells, T_cell_co-stimulation, T_cell_co-inhibition, pDCs MHC_class_I, Inflammation-promoting, Cytolytic_activity, Check point, CD8+_T_cells, Macrophages down, and CCR, were significantly enriched in IPAH compared with normal samples. These results suggest that complex immune components are involved in the pathology of IPAH (Fig. 5).

### Competitive endogenous RNA (ceRNA) networks based on marker genes

**Fig. 6:**
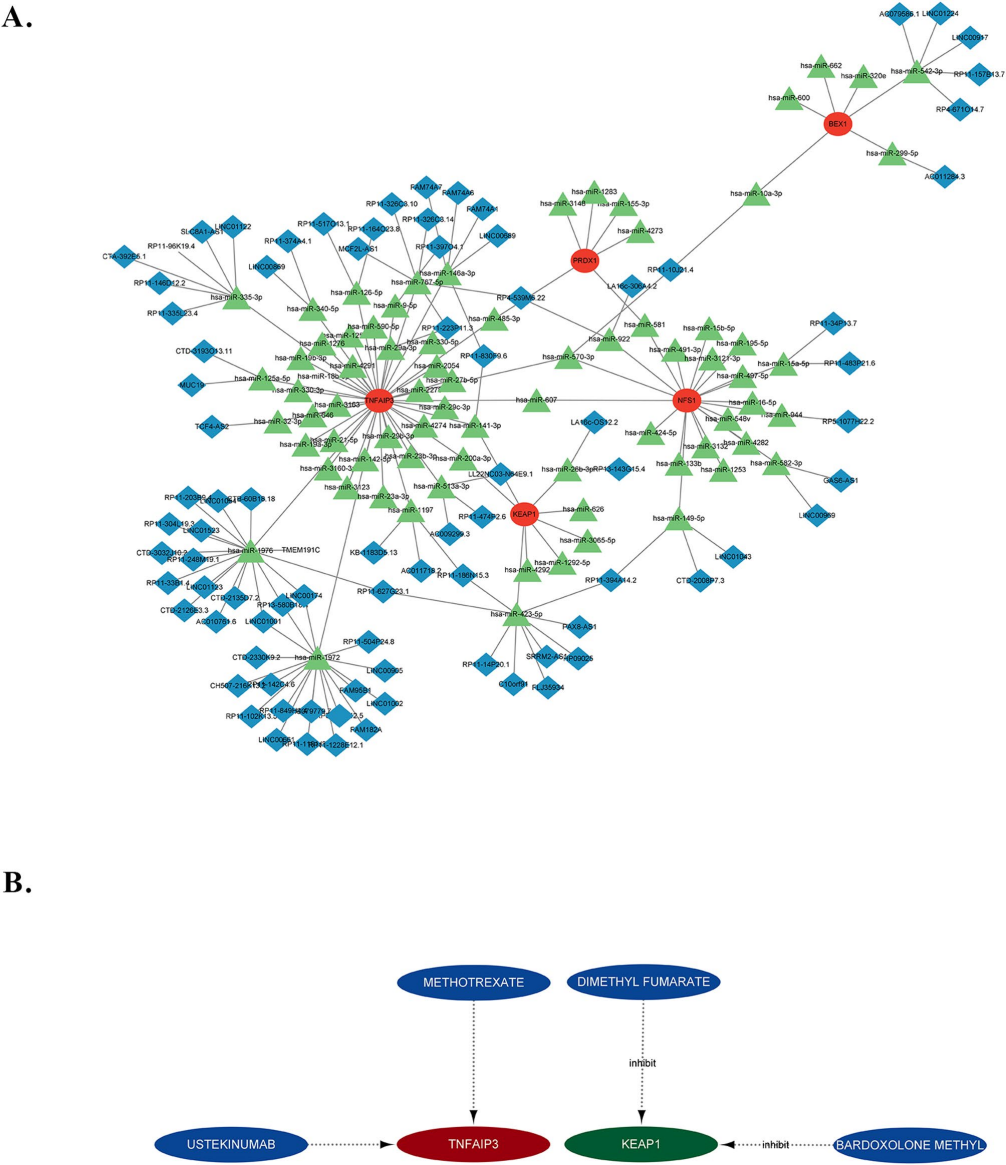
A. A ceRNA network, including 166 nodes (5 marker genes, 81 miRNAs and 86 lncRNAs), was constructed based on the 6 marker genes. **B**. The DGIdb was used to identify drugs possibly targeting marker genes, showing that only two genes have the target drugs.

Next, a ceRNA network with 166 nodes (5 marker genes, 81 miRNAs, and 86 lncRNAs) was constructed based on the 6 marker genes (Fig. 6A). We observed that *TNFAIP3* was regulated by the most predicted miRNAs and lncRNAs. *PRDX1* was regulated by hsa-miR-3148, hsa-miR-581, hsa-miR-155-3p, hsa-miR-485-3p, and hsa-miR-1283 without lncRNAs, whereas seven lncRNAs binding to hsa-miR-600, hsa-miR-10a-3p, hsa-miR-320e, hsa-miR-662, hsa-miR-542-3p, and hsa-miR-4273 may regulate the expression of *BEX1*. In addition, 11 lncRNAs could regulate the expression of *KEAP1* by competitively binding to hsa-miR-1292-5p, hsa-miR-141-3p, hsa-miR-423-5p, hsa-miR-626, hsa-miR-200a-3p, hsa-miR-3065-5p, hsa-miR-4292, and hsa-miR-26b-3p. Conversely, eight lncRNAs could competitively bind hsa-miR-4282, hsa-miR-16-5p, hsa-miR-944, hsa-miR-548v, hsa-miR-581, hsa-miR-15b-5p, hsa-miR-491-3p, hsa-miR-922, hsa-miR-1253, hsa-miR-195-5p, hsa-miR-15a-5p, hsa-miR-149-5p, hsa-miR-133b, hsa-miR-424-5p, hsa-miR-3121-3p, hsa-miR-497-5p, hsa-miR-607, hsa-miR-582-3p, hsa-miR-3132, and hsa-miR-570-3p to regulate the expression of *NFS1*. Furthermore, both *TNFAIP3* and *NFS1* expression were regulated by hsa-miR-607 and hsa-miR-570-3p, which could also regulate the expression of *BEX1*.

### Prediction of marker gene-targeted drugs

The DGIdb was used to identify drugs targeting marker genes, revealing that only two genes had associated target drugs (Fig. 6B). Bardoxolone methyl and dimethyl fumarate are inhibitors of *KEAP1*, whereas ustkinumab and stekinumab are unidentified inhibitors of *TNFAIP3*. Notably, drugs targeting *BEX1*, *PRDX1*, *NFS1*, or *MEG3* have not been identified to date.

### Marker gene expression in the validation set

**Fig. 7:**
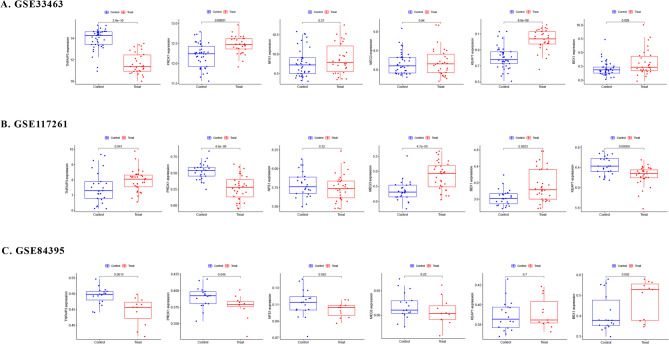
A. GSE33463, **B**. GSE117261, **C**. GSE84395 were employed as validation sets to verify the expression of marker genes.

Validation was performed using GSE117261, GSE33463, and GSE84395 to confirm the expression patterns of marker genes. Initially, we assessed marker gene expression trends in the GSE117261 dataset, including 31 IPAH and 25 healthy tissue samples. *KEAP1*, *BEX1*, *PRDX1*, *TNFAIP3*, and *MEG3* in the GSE117261 dataset exhibited expression trends similar to those observed in the GSE48149 dataset. Subsequently, compared to the GSE48149 dataset, the GSE33463 dataset, consisting of 30 IPAH and 41 healthy PBMC samples, was analyzed. It revealed a similar expression level trend for *BEX1*, contrasting with the patterns of *KEAP1*, *PRDAX1*, and *TNFAIP3*. Additionally, we examined marker gene expression levels in GSE84395, including 11 IPAH and 18 healthy PEC samples. The expression trends of *PRDX1* remained consistent with the GSE48149 dataset, whereas the expression of *TNFAIP3* was lower in IPAH (Fig. 7).

### Validation in a single-cell dataset

**Fig. 8:**
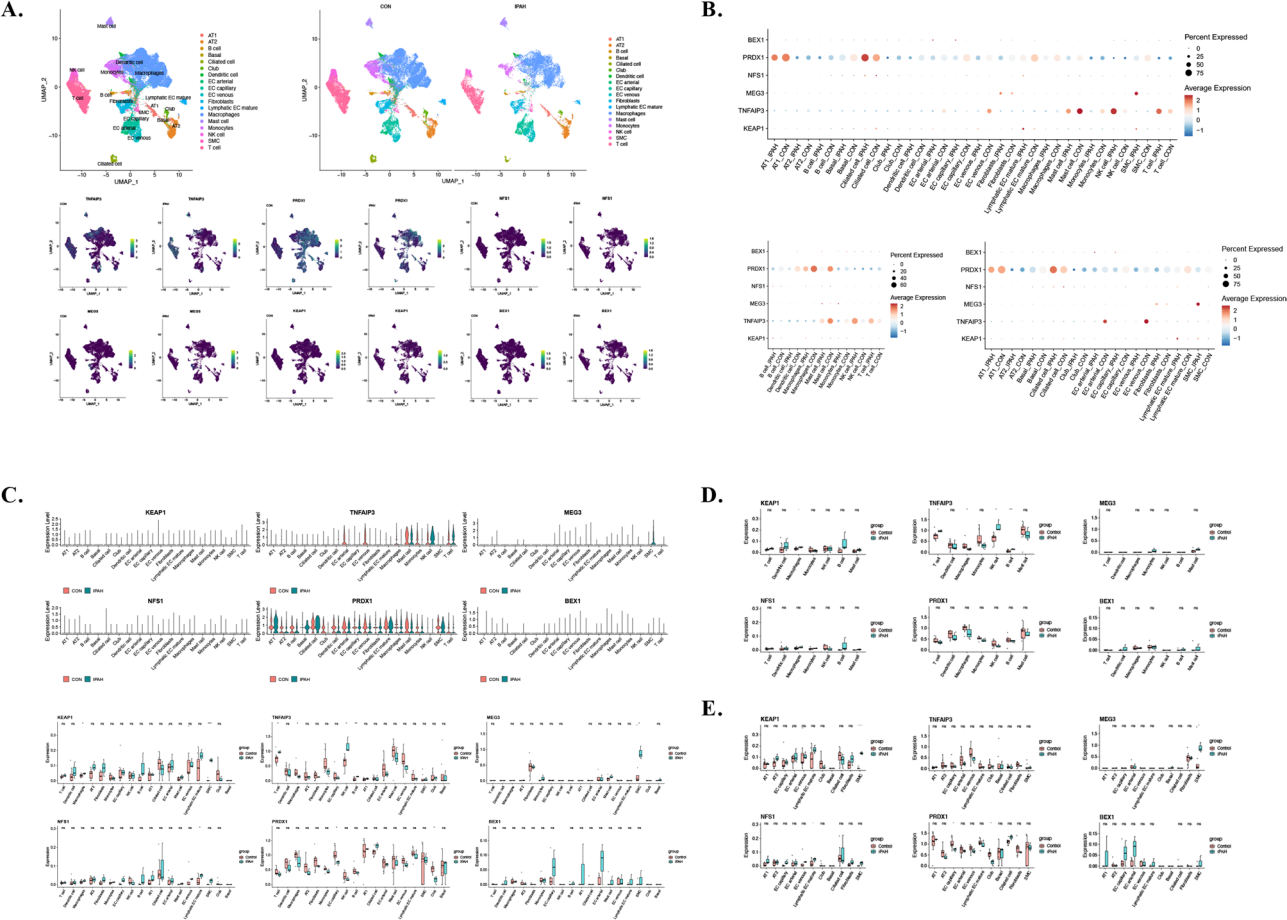
The single-cell dataset GSE169471 was employed to test the marker gene expression in different cells. **A**. Expression levels of six marker genes—*KEAP1*,* BEX1*,* PRDX1*,* TNFAIP3*,* NFS1*, and *MEG3*—were marked in respective cell types. Dot plot **(B)**, violin plot and boxplot **(C)** showing the expression levels of the six marker genes in respective cell types. **D**. Expression levels of the six marker genes in immune cells. E. Expression levels of the six marker genes in other cells.

The single-cell dataset GSE169471, which included three patients with IPAH and eight healthy tissue samples, was used to assess marker gene expression in various cell types. Following quality control, normalization, data scaling, and dimensionality reduction, cells were classified into 18 clusters: AT1, AT2, B cell, Basal, Ciliated cell, Club, Dendritic cell, EC arterial, EC capillary, EC venous, Fibroblasts, Lymphatic EC mature, Macrophages, Mast cells, Monocytes, NK cells, SMC, and T cells. Subsequently, six marker genes—*KEAP1*, *BEX1*, *PRDX1*, *TNFAIP3*, *NFS1*, and *MEG3—*were identified in the respective cell types. Notably, compared to normal tissues, smooth muscle cells in IPAH exhibited high expression of *MEG3* and *KEAP1*. Furthermore, *KEAP1* was predominantly expressed in macrophages. Among immune cells, T cells, macrophages, NK cells, and B cells exhibited associations with *TNFAIP3*, which was highly expressed in IPAH. Similar to GSE84395, *PRDX1* displayed low expression levels in capillary endothelial cells (Fig. 8).

## Discussion

IPAH is a rare and life-threatening condition. Despite advancements in understanding the disease, its precise molecular mechanisms remain unclear due to the multifaceted interplay between genetic, environmental, and cellular factors. Previous research has underscored the intricate association between ferroptosis and various cardiovascular diseases, including cardiomyopathy, myocardial infarction, ischemia/reperfusion injury, and heart failure^36^.Ferroptosis in the systemic vasculature leads to endothelial cell dysfunction and promotes the development of atherosclerosis in mice. Notably, this effect can be attenuated by ferrostatin-1 treatment^37^ This association extends to the pulmonary domain, where the pulmonary vasculature plays a pivotal role in ferroptosis due to the unique composition of oxygenated blood, which is rich in polyunsaturated fatty acids and iron ions. Initially elucidated within oncological frameworks, ferroptosis has emerged as a significant contributory factor in the pathogenesis of various pulmonary disorders, including chronic obstructive pulmonary disease, acute lung injury, and pulmonary fibrosis. Current scientific inquiries are vigorously directed towards identifying and characterizing ferroptosis biomarkers across morphological, biochemical, proteomic, and genomic domains. However, identifying reliable gene biomarkers and the precise regulatory intricacies linked to ferroptosis in IPAH remain a formidable challenge in medical research. Our comprehensive analysis explored potential ferroptosis-related biomarkers and therapeutic targets in IPAH, offering valuable insights into its complex pathophysiology and presenting novel diagnostic and therapeutic avenues.

In our study, we identified 27 DE-FRGs that exhibited significant differences between IPAH and normal samples, comprising 15 downregulated and 12 upregulated genes. The GO enrichment analysis highlighted significant associations of DE-FRGs with CC (“focal adhesion,” “cell–substrate junction,” “membrane raft”), BP (“cellular response to chemical stress,” “cellular response to peptidyl-serine phosphorylation,” “peptidyl-serine modification”), and MF (“RNA polymerase II-specific DNA-binding transcription factor binding,” “DNA-binding transcription factor binding,” “phosphoprotein binding”). Notably, focal adhesion, which is critical for cell adhesion and migration within the matrix, may regulate interactions between pulmonary vascular endothelial cells and their environment during remodeling^38^. Maintenance of cell–substrate junctions is crucial for intercellular communication and tissue integrity, influencing communication between pulmonary vascular endothelial cells and subsequently affecting vascular wall structure, function, remodeling, and narrowing. These cellular alterations likely contribute significantly to pulmonary vascular pathology, influencing the process of vascular remodeling significantly^39^. Concurrently, Reactome pathway analyses indicated a notable enrichment in pathways such as fluid shear stress, atherosclerosis, hepatocellular carcinoma, and chemical carcinogenesis, specifically related to reactive oxygen species. The restructuring of blood vessels caused by fluid shear stress may be associated with the generation of detrimental substances that are potentially linked to atherosclerosis via iron-mediated pathways. These pathways may induce lipid damage, affect protein carbonylation, and contribute to pulmonary vascular remodeling^40^. This evidence suggests that DE-FRGs play pivotal roles in the regulation of autophagy, cytokines, kinases, and immune cells, potentially contributing to critical effects on IPAH pathogenesis.

This analysis identified six DE-FRGs as potential diagnostic markers of IPAH using robust machine learning. Validation across independent datasets (GSE117261 and GSE84395) generally supported their potential, although discrepancies surfaced in GSE33463, particularly evident in the varied expression of *KEAP1*, *PRDX1*, and *TNFAIP3*. Among the DE-FRGs, we suggest that *PRDX1* is linked to endothelial dysfunction and that *TNFAIP3* is associated with immune responses, serving as a potential diagnostic marker for IPAH.

Pulmonary vascular remodeling, a hallmark pathological feature of PAH, arises from the dysregulated interactions of multiple vascular cell populations, with dysfunctional endothelial cells (ECs) serving as both initiators and amplifiers of disease progression^41^. Factors such as hypoxia, inflammation, and shear stress can damage pulmonary vascular ECs, especially pulmonary artery ECs (PAECs) and pulmonary arterial endothelial cell (PAEC) dysfunction, characterized by mitochondrial impairment and altered iron and lipid metabolism, creates a pro-ferroptotic microenvironment in PAH^42^. In studies on monocrotaline (MCT)-induced PAH in rats, Xie et al. found weakened PAEC activity, along with increased levels of ferroptosis-related proteins in PAECs^15^. The ferroptosis inhibitor, ferristatin-1, was able to mitigate the progression of MCT-induced pulmonary vascular remodeling by downregulating these markers^42^. This hypothesis is supported by the observation that small molecule and genetic interventions that suppress ferroptosis reduce PAH severity in rodents^14,37,42^. Furthermore, in vitro studies suggest that ferroptosis in PAECs can modulate the progression of PAH through the HMGB1/TLR4/NLRP3 inflammasome signaling pathway, indicating a relationship between ferroptosis and EC dysfunction^13^. Its pathogenicity primarily originates from a cascade of oxidative stress-induced lipid peroxidation, ultimately compromising the structural integrity of the pulmonary vasculature^43^.These studies suggest that inhibiting ferroptosis may reduce pulmonary vascular remodeling due to EC dysfunction, serving as a potential target for PAH treatment. In our study, *PRDX1*, consistent downregulation was observed in the IPAH group compared with healthy controls, except for GSE33463. This trend was reinforced by findings from single-cell sequencing analysis (GSE169471), which demonstrated diminished *PRDX1* expression in IPAH capillary endothelial cells compared with healthy tissue, potentially implicating its involvement in endothelial dysfunction, a key aspect of IPAH pathology.

The Peroxiredoxins (PRDXs) are a family of six thiol-dependent peroxidases (PRDX1-PRDX6)^44^. PRDXs can catalyze the reduction of hydroperoxide and hydrogen peroxide and have been implicated as antioxidant signaling proteins^45,46^.Previous research has shown that PRDXs are important modulators of physiological and pathophysiological cardiovascular events. PRDX3 overexpression prevents cardiac hypertrophy and HF by reducing oxidative stress and restoring mitochondrial function. PRDX2 decressed expression during cardiac hypertrophy and may be a potential therapeutic target for the treatment of cardiac hypertrophy^47,48^. Ping Dai et al. Found that PRDX6 was expressed in PAECs and was significantly decreased in MCT-induced PAH. They suggested that PRDX6 mediates PAEC ferroptosis through HMGB1 release and subsequent activation of TLR4/NLRP3 inflammasome signalling^14^. PRDX1is a typical 2-cysteine (Cys) peroxiredoxin. Its primary function is to catalyze reduction reactions, including the conversion of hydrogen peroxide (H2O2) to water, and to act as a multifunctional antioxidant^49^. PRDX1 functions as an antioxidant factor and redox signaling protein that regulates various metabolic pathways and lysosomal processes to mitigate oxidative stress and influence oxidant sensitivity^50,51^. *PRDX1* plays an important role in countering increased endothelial activation^52^. Previous studies have shown that PRDX1 could resist oxidation, protect cells from oxidative damage, and improve cardiac hypertrophy^49^. This evidence suggests a potential protection role for PRDX1 in IPAH pathology.

The intricate pathogenesis of PAH remains under ongoing investigation without definitive clarity. Recent years of research in both preclinical and clinical studies within the PAH field have increasingly emphasized the involvement of inflammation and immunity in its progression^53^. Notably, reports have highlighted the presence of circulating autoantibodies targeting endothelial cells and fibroblasts in a substantial percentage (10–40%) in patients diagnosed with IPAH and SSc-PAH. These findings suggest a potential association between autoimmunity and the development of pulmonary vascular lesions^54,55^. We used CIBERSORT to detect elevated expression of activated NK cells, macrophages M1, and activated T cells CD4 memory in patients with IPAH. Moreover, Pearson’s correlation analysis revealed positive correlations between neutrophils and *TNFAIP3* and negative correlations between macrophages M0 and *PRDX1*. In our single-cell validation dataset, immune cell types, such as T cells, macrophages, NK cells, and B cells, were associated with *TNFAIP3*, which exhibited high expression levels in IPAH. Similar to PRDX1, TNFAPI3 exhibited elevated expression levels in the IPAH cohort, except in the validation dataset GSE33463. TNFAIP3, an anti-inflammatory protein, plays a significant role in the immune response and cellular apoptosis^56^. In contrast to other organ systems, the lungs demonstrate comparatively elevated levels of *TNFAIP3* mRNA expression, with detectable protein levels observed in whole-lung homogenates of rodents^57^. Dysfunctions in TNFAIP3 have implications in various autoimmune conditions, such as Crohn’s disease, asthma, and chronic obstructive pulmonary disorder^58^. Subsequent pathway analysis demonstrated that TNFAIP3 upregulation influenced diabetes-related pathways and immune responses. Most chronic inflammatory autoimmune diseases are associated with reduced TNFAIP3 expression and continuous NF-kB activation^58^. Paradoxically, high TNFAIP3 levels have been associated with disease promotion, suggesting a cell- and organ-specific approach to TNFAIP3 augmentation to prevent adverse effects and induce metabolic benefits^59^. GSE33463, containing 30 IPAH and 41 healthy PBMC samples, showed lower expression of *TNFAIP3*. Moreover, in neurodegenerative diseases associated with systemic inflammation, basal *TNFAIP3* mRNA expression in peripheral blood lymphocytes and monocytes is significantly lower than in healthy individuals^60^. These findings are consistent with our expression analysis of TNFAIP3 in IPAH, where upregulation in macrophages was observed, indicating its potential role in inflammatory processes that drive iron dysregulation and ferroptosis in IPAH.

In addition, KEAP1, NFS1, MEG3, and BEX1, though not as prominently featured in the single-cell analysis, are known to be involved in oxidative stress regulation, iron metabolism, and cell cycle regulation, all of which are processes critical to ferroptosis. KEAP1, for instance, regulates oxidative stress responses by controlling NRF2 activity. In the context of IPAH, downregulation of KEAP1 could lead to enhanced oxidative stress, further exacerbating ferroptosis in endothelial cells and smooth muscle cells, thereby contributing to vascular remodeling. Similarly, NFS1 is involved in the synthesis of iron-sulfur clusters, and its downregulation in IPAH may disrupt iron homeostasis, promoting ferroptosis in susceptible cells. MEG3 and BEX1 are also involved in regulating cell cycle and apoptosis, processes that are crucial in maintaining vascular integrity in IPAH. As ferroptosis is tightly linked with these cellular processes, alterations in the expression of these genes may have profound implications for disease progression.

The establishment of a comprehensive ceRNA network centered on marker genes sheds light on the intricate regulatory interactions among miRNAs and lncRNAs. TNFAIP3 has emerged as a central node influenced by numerous miRNAs and lncRNAs. However, PRDX1 is specifically regulated by distinct miRNAs, excluding lncRNA involvement. Furthermore, the identification of drugs targeting marker genes has uncovered limited options, with no drugs identified for targeting PRDX1, highlighting the scarcity of pharmaceutical options addressing these genes in the context of IPAH treatment.

Despite the comprehensive nature of our analysis, several limitations warrant consideration. Firstly, the sample sizes in individual datasets were relatively small, potentially influencing the robustness and generalizability of our findings. Additionally, the inherent heterogeneity across IPAH subtypes may introduce variability in the observed gene expression patterns. Furthermore, the use of diverse cell and tissue sources across datasets could contribute to variations in the results. While our bioinformatic approach provides valuable insights, experimental validation is necessary to confirm the functional relevance of the identified biomarkers. Lastly, the need for further research is emphasized to validate the potential drug targets for their clinical applicability. These limitations underscore the importance of cautious interpretation and future investigations to refine our understanding of ferroptosis-related mechanisms in IPAH.

## Conclusions

In summary, our exploration, facilitated by a comprehensive bioinformatic analysis, delved into the ferroptosis genes within IPAH. We identified six DE-FRGs—*KEAP1*, *TNFAIP3*, *MEG3*, *NFS1*, *PRDX1*, *BEX1*—among which *PRDX1* and *TNFAIP3* exhibited stable expression across multiple datasets. These genes are implicated in critical pathways, including cell cycle regulation, immune response modulation, and vascular constriction, indicating their involvement in IPAH development. Furthermore, the identification of potential drug targets, such as the TNFAIP3-targeting ustkinumab, offers promising avenues for therapeutic interventions in IPAH. These findings propose PRDX1 and TNFAIP3 for future use in predictive diagnostics, prevention, patient stratification, and ustkinumab as the personalized medicine for IPAH. Nevertheless, further research must be conducted to validate potential drug targets for clinical applicability.

## Data Availability

The datasets generated and/or analysed during the current study are available in GSE48149 repository, https://www.ncbi.nlm.nih.gov/geo/query/acc.cgi?acc=GSE48149, GSE117261 repository, https://www.ncbi.nlm.nih.gov/geo/query/acc.cgi?acc=GSE117261, GSE84395 repository, https://www.ncbi.nlm.nih.gov/geo/query/acc.cgi?acc=GSE84395, GSE33463 repository, https://www.ncbi.nlm.nih.gov/geo/query/acc.cgi?acc=GSE33463, GSE169471 repository, https://www.ncbi.nlm.nih.gov/geo/query/acc.cgi?acc=GSE169471.
